# Deletion of the muscle enriched lncRNA Oip5os1 induces atrial dysfunction in male mice with diabetes

**DOI:** 10.14814/phy2.15869

**Published:** 2023-12-06

**Authors:** Aowen Zhuang, Yanie Tan, Yingying Liu, Christine Yang, Helen Kiriazis, Kyah Grigolon, Shannen Walker, Simon T. Bond, Julie R. McMullen, Anna C. Calkin, Brian G. Drew

**Affiliations:** ^1^ Baker Heart & Diabetes Institute Melbourne Victoria Australia; ^2^ Central Clinical School Monash University Melbourne Victoria Australia; ^3^ Baker Department of Cardiometabolic Health University of Melbourne Melbourne Victoria Australia

**Keywords:** atrial dysfunction, cardiomyopathy, diabetes, non‐coding RNA

## Abstract

Long ncRNAs (lncRNAs) have been shown to play a biological and physiological role in various tissues including the heart. We and others have previously established that the lncRNA *Oip5os1* (*1700020I14Rik*, OIP5‐AS1, Cyrano) is enriched in striated muscles, and its deletion in mice leads to defects in both skeletal and cardiac muscle function. In the present study, we investigated the impact of global *Oip5os1* deletion on cardiac function in the setting of streptozotocin (STZ)‐induced diabetes. Specifically, we studied male WT and KO mice with or without diabetes for 24 weeks, and phenotyped animals for metabolic and cardiac endpoints. Independent of genotype, diabetes was associated with left ventricular diastolic dysfunction based on a fall in E'/A' ratio. Deletion of *Oip5os1* in a setting of diabetes had no significant impact on ventricular function or ventricular weight, but was associated with left atrial dysfunction (reduced fractional shortening) and myopathy which was associated with anesthesia intolerance and premature death in the majority of KO mice tested during cardiac functional assessment. This atrial phenotype was not observed in WT diabetic mice. The most striking molecular difference was a reduction in the metabolic regulator ERRalpha in the atria of KO mice compared with WT mice. There was also a trend for a reduction in Serca2a. These findings highlight *Oip5os1* as a gene of interest in aspects of atrial function in the setting of diabetes, highlighting an additional functional role for this lncRNA in cardiac pathological settings.

## 1 INTRODUCTION

Non‐coding RNAs (ncRNAs) are transcribed in vast quantities from the genome often via tightly regulated mechanisms; however, ncRNAs do not locate to the ribosome and therefore are unable to code for peptides or proteins. Their size and function vary greatly, but most have co‐operative activities through their target molecules, which primarily work to fine‐tune signaling cascades and biological responses. Long non‐coding RNAs (lncRNAs) are ncRNAs that are >200 nucleotides in length, and are often topographically similar to protein coding mRNAs (Kashi et al., 2016). They have been demonstrated to have an extensive array of functions that impact on processes such as interacting with transcriptional machinery, forming structural scaffolds, or acting as RNA sponges (Mallory & Shkumatava, 2015; Ulitsky & Bartel, 2013). Although lncRNAs are a comparatively recently described class of ncRNA, data from the past decade have demonstrated that they are promising targets for therapeutic and biomarker applications.

With the molecular cascades that regulate cardiac commitment and development being an intense area of interest, the discovery of lncRNAs has spawned a new area of investigation in cardiac biology (McMullen & Drew, 2016). Indeed, lncRNAs have since been shown to be involved in most facets of cardiac commitment, development, and function. lncRNAs such as Braveheart (*bvht*) (Klattenhoff et al., 2013), *Fendrr*, *SRA*, and *Novlnc6*, (Colley & Leedman, 2011; Grote et al., 2013; Ounzain et al., 2015) have all been demonstrated to affect the activity of lineage‐specific transcriptional pathways. Moreover, lncRNAs have also been associated with cardiac pathology including *MIAT, LIPCAR, Mhrt*, and *CHRF* (Han et al., 2014; Ishii et al., 2006; Kumarswamy et al., 2014; Wang et al., 2014). Of these, MyHeart (*Mhrt*) was a particularly interesting example, which is reduced in the setting of cardiac hypertrophy, but demonstrated improved cardiac function when reconstituted, providing evidence that lncRNAs may have direct therapeutic relevance in the setting of heart failure (Han et al., 2014).

Our group recently explored the role of the lncRNA *1700020I14Rik* (also known as *Oip5os1*, *OIP5‐AS1*, and Cyrano; which we refer to hence forth as *Oip5os1* given we are studying the mouse variant) in a setting of pressure overload‐induced cardiomyopathy and heart failure in mice. Deletion of *Oip5os1* was associated with worse cardiac pathology, which was more severe in female than male mice. The underlying mechanisms were shown to involve dysregulated metabolism in the heart, particularly relating to activity of transcription factors that regulate mitochondrial activity (Zhuang, Calkin, et al., 2021). Since diabetes is known to drive changes in cardiac metabolism, the goal of this study was to assess whether loss of *Oip5os1* exacerbated STZ‐induced cardiomyopathy. We demonstrate that deletion of *Oip5os1* in a setting of diabetes had no impact on left ventricular diastolic function but was associated left atrial dysfunction and premature death in a significant percentage of mice during echocardiography. These chamber‐specific findings were unexpected, highlighting the complex regionality of cardiac dysfunction, while shedding light on the contribution of *Oip5os1* to atrial function in the setting of cardiac disease.

## 2 METHODS

### 2.1 Animals cohorts and diabetes induction

*Oip5os1* knockout (KO) mice were generated in our laboratory on a C57BL/6J background with the Australian Phenomics Network as previously described (Zhuang, Calkin, et al., 2021). Mice were bred as heterozygous parental lines and cohorts of littermate wildtype (WT), and KO mice were generated for subsequent experiments. Diabetes was induced in male mice at approximately 6–7 weeks of age by five consecutive days of intraperitoneal injection of low‐dose (55 mg/kg) streptozotocin (STZ; Sigma S0103) in citrate buffer. Control animals received the same injections, but of citrate buffer only. It was not possible to use female mice in the current study due to the known resistance of females to STZ using this protocol. Fasting blood glucose (FBG) was tested once weekly on these mice over the subsequent 4 weeks, after which animals with a FBG <15 mmol/L were considered to not have diabetes. Mice from the STZ‐treated cohorts that presented without diabetes (~20%) were subsequently excluded from the study. All animals were housed at 22°C on a 12 h light/dark cycle with ad libitum access to food (standard rat and mouse chow, Specialty feeds, Australia) and water. At the conclusion of the study, mice were fasted for 4–6 h and then euthanized by an overdose of ketamine‐xylazine (Pfizer) before plasma and tissues were collected then either used fresh, or flash frozen. All experiments were approved by the Alfred Research Alliance (ARA) Animal Ethics Committee and performed in accordance with the NHMRC of Australia guidelines for the care and use of laboratory animals.

### 2.2 Echocardiography

To obtain measures of LV and LA function, echocardiography was performed as previously described (Bernardo et al., 2022). Briefly, mice were anesthetized (ketamine/xylazine/atropine, KXA: 80/8/0.96 mg/kg, i.p.; Troy laboratories and Pfizer) at ~22 weeks following STZ administration, and echocardiography was performed using a Vevo 2100 High Frequency Ultrasound System (Visual Sonics). Once mice were anesthetized, fur was removed using a depilatory cream, wiped clean and mice then placed on a Visualsonics handling platform in a supine position. Acoustic coupling gel was placed on the chest area and images acquired using a MS550D transducer. Core temperature was monitored using a rectal probe and maintained at the physiological level (36–37°C). Evaluation of diastolic function was performed by echocardiography using measurement of transmitral flow parameters including the early (E) and late (A) diastolic filling velocities, the E/A ratio, from an apical four chamber view with pulsed wave Doppler. Tissue Doppler imaging was also performed to obtain early (e') and late (a') diastolic mitral annular velocity (e'/a' ratio) and E/e' ratio. LA dimension at systole (LAs) and diastole (LAd) was measured at the level of the aortic root from the parasternal long‐axis view. LA fractional shortening was assessed as: *[[LAd‐LAs]/LAd] × 100%*. At completion of echocardiography, mice were administered atipamezole (0.2 mg/kg, subcutaneously; Zoetis) to aid with recovery post KXA anesthesia. All echocardiography imagery was acquired by a single operator and analyzed blinded offline using the Vevo Lab Software (Version 3.2.6, Visual Sonics). All data were independently validated (Donner et al., 2018).

### 2.3 Glucose tolerance and blood glucose testing

Oral glucose tolerance tests (oGTTs) were conducted at the indicated time points as previously described (Bond, Zhuang, et al., 2021; Zhuang, Yang, et al., 2021). Briefly, mice were fasted for 5–6 h prior to blood glucose measures being taken at baseline via a drop of blood from the tail vein using a glucometer (Accu Check Performa, Roche Diabetes Care). Following this, mice were given glucose via oral gavage (2 g/kg lean mass) and blood glucose testing was conducted at 15, 30, 45, 60, 90, and 120 min post glucose gavage.

### 2.4 Body composition by EchoMRI

Body composition in live animals was measured using the 4‐in‐1 EchoMRI (Houston, TX, USA). One at a time, each conscious mouse was placed in the chamber, with body weight, lean mass, and fat mass recorded at 22 weeks, as previously described (Bond, King, et al., 2021; Zhuang, Yang, et al., 2021).

### 2.5 Tissue weights and heart‐specific parameters

At the end of the study, tissues were collected and weighed before being snap frozen in liquid nitrogen. For the heart, whole heart wet weight was determined before dissection of atria (together) and right ventricle (RV). RV and LV were weighed separately, and atria weight was estimated by subtracting LV and RV weights (combined) from total heart weight. Tibias were trimmed of tissue and cleaned in 1 M HCl for 30 min at 55°C before being washed in PBS, dried and measured using high precision calipers.

### 2.6 Quantitative PCR (qPCR)

RNA for qPCR analysis was isolated from tissues as previously described (Bond, Kim, et al., 2019; Bond, Moody, et al., 2019). Briefly, tissues were homogenized in RNAzol reagent (in‐house preparation) and precipitated using isopropanol (Merck). cDNA was generated from 1 μg of RNA using MMLV reverse transcriptase (Invitrogen) according to the manufacturer's instructions. qPCR was performed on 10 ng of cDNA using the SYBR‐green method on a QuantStudio 7 Flex (ThermoFisher Scientific) using gene‐specific primers (IDT Technologies) (see Table 1 for Primer Details). Quantification of a given gene was expressed by the relative mRNA level compared with control, which was calculated after normalization to the housekeeping gene 36B4 (*Rplp0*) or Cyclophilin A (*Ppia*) using the delta‐CT method. Primers were designed to span exon‐exon junctions where possible and were tested for specificity using BLAST (Basic Local Alignment Search Tool; National Centre for Biotechnology Information). Amplification of a single amplicon was estimated from melt curve analysis, ensuring only a single peak and an expected temperature dissociation profile were observed.

**TABLE 1:** Primer sequences for qPCR.

Gene name	Primer sequence	
	Forward (5′ – 3′)	Reverse (5′ – 3′)
*Oip5os1*	CAACACTTGACACCCTATCC	CACCACTCTCAAGTCGATTAC
*Atp2a2 (SERCA2)*	AATATGAGCCTGAAATGGGC	TCAGCAGGAACTTTGTCACC
*Myh6*	CTCTTCAGCAGCGGTTTGAT	AAGATAGTGGAACGCAGGGA
*Myh7*	AGCATTCTCCTGCTGTTTCC	GAGCCTTGGATTCTCAAACG
*Esrra (ERRα)*	GTGGCCTCTGGCTACCACTA	CGCTTGGTGATCTCACACTC
*Nrf2*	CATGATGGACTTGGAGTTGC	CCTCCAAAGGATGTCAATCAA
*Ppargc1a (PGC1α)*	TGAGGACCGCTAGCAAGTTT	TGAAGTGGTGTAGCGACCAA
*Sdha*	TGGACCCATCTTCTATGC	TACTACAGCCCCAAGTCT
*Ndufs1*	CACTCGTTCCACCTCAGCTA	GACGGCTCCTCTACTGCCT
*Nppa (ANP)*	GGGGGTAGGATTGACAGGAT	AGGGCTTAGGATCTTTTGCG
*Nppb (BNP)*	ACAAGATAGACCGGATCGGA	AAGAGACCCAGGCAGAGTCA
*Col3a1*	GGGAATGGAGCAAGACAGTCTT	TGCGATATCTATGATGGGTAGTCTCA
*Col1a1*	ACATGTTCAGCTTTGTGGACC	GGTTTCCACGTCTCACCATT
*Rplp0*	ACCCTGAAGTGCTCGACATC	ATTGATGATGGAGTGTGGCA
*Ppia*	AGCCAAATCCTTTCTCTCCAG	CACCGTGTTCTTCGACATCA

### 2.7 Data inclusion/Exclusion criteria

For animal phenotyping data, individual data points were excluded if they were technically implausible or a methodological error had resulted in a spurious outcome. Animals were excluded from diabetes analyses if their FBG was <15 mmol/L at the 4 week time point post‐STZ. Analyses from animals were excluded if animals were found dead or unwell, or technical/analytical problems were identified (compromised RNA, failed analysis, improper tissue collection, equipment failure). For echocardiography, data were excluded if heart rates were < 350 bpm or animals demonstrated compromised health from chronic diabetes. See below for further exclusions relating to STZ and echocardiography that impacted on group numbers and statistics.

### 2.8 Statistical analysis and experimental framework

All animal and laboratory data underwent blinding and randomization at time of collection and during technical analysis. Sample size for each cohort were determined from previous studies in our research group which investigated STZ‐induced diabetes based on the equation: (α × [*SD*×*SD*] × β)/(*Diff*×*Diff*) where SD = standard deviation, Diff = difference from the mean, α = significance level (i.e., 0.05), and β = power. The main endpoint of interest was heart function in the setting of diabetes, and therefore, we prioritized this for numbers per group. Accordingly, in the original study we had *N* = 6 for WT control, *N* = 16 for WT diabetic, *N* = 6 for KO control, and *N* = 16 for KO diabetic. As anticipated with STZ treatment, some mice (two; both KO) receiving STZ did not convert to full diabetes at 4 weeks post‐injection and had to be excluded. Final numbers that completed the 24 week experiment were *N* = 6 for WT control, *N* = 16 for WT diabetic, *N* = 6 for KO control, and *N* = 14 for KO diabetic. It should be noted that numbers are lower than anticipated for some parameters, particularly cardiac function (echocardiography) due to the unexpected loss of KO diabetic mice during the final echocardiography under anesthesia, and the subsequent exclusion of this procedure on remaining mice due to ethical reasons (meaning echo was not performed for *n* = 7 mice with diabetes and *n* = 3 control mice). Data were expressed as mean ± standard deviation of the mean (SD), unless otherwise stated. All statistical analyses of animal and laboratory based experiments were performed using PRISM7 software. Normally distributed data were analyzed by unpaired two‐tailed *t*‐test to test for significant differences between the means in either direction of unrelated datasets between WT and KO animals, unless otherwise stated. In these analyses, a *p*‐value of *p* < 0.05 was considered statistically significant.

## 3 RESULTS

### 3.1 Glucose parameters and body composition in *Oip5os1* KO mice with and without diabetes

**FIGURE 1:**
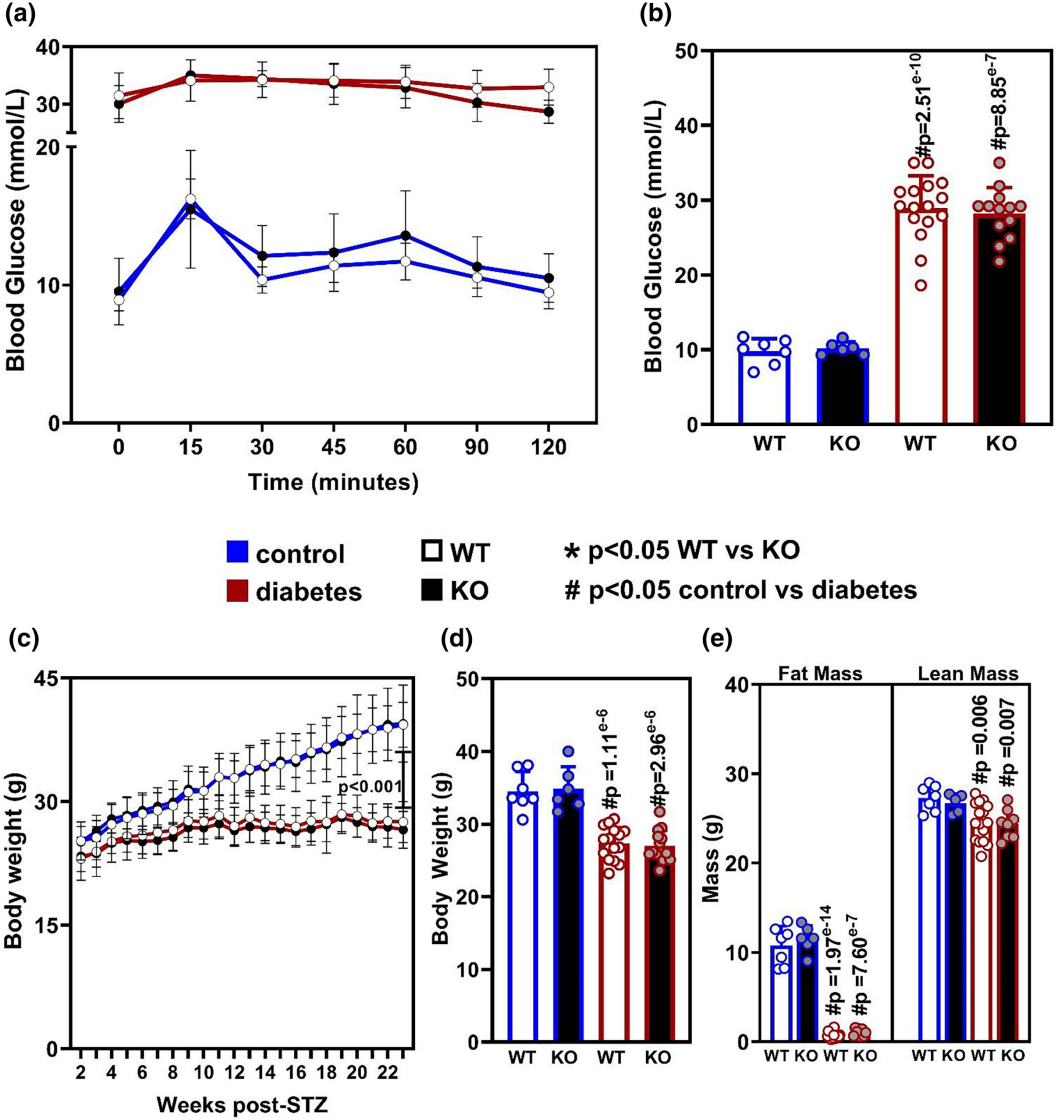
Glucose Homeostasis and Body Weight/Composition of WT and KO mice with and without diabetes. (a) Blood glucose measurements during oral glucose tolerance testing in WT (open circles), KO (black circles) animals with diabetes (red lines) and without diabetes (blue lines) at study end. (b) Basal fasting blood glucose levels at study end. (c) Weekly body weights of all four cohorts across the 6‐month experimental time course. (d) Body weights of each cohort at study end. (e) Body composition including fat mass and lean mass at study end as determined by EchoMRI. *n* = 7–15/group, mean ± SD, *p < 0.05 WT versus KO, # *p* < 0.05 Control versus Diabetes as determined by non‐parametric one‐way ANOVA with multiple comparisons correction (Dunnet's).

We have previously described a global *Oip5os1* null mouse generated by our laboratory, which demonstrated no detectable expression of *Oip5os1* in all tissues analyzed (Zhuang, Calkin, et al., 2021). In brief, the entire genomic location (~13 kb) of this gene was removed in the KO mice, covering everything from the transcriptional start site, all exons and introns, and ending at the final codon of the last exon. All promoter and enhancer regions were left intact. Accordingly, this is a complete KO of all possible transcripts of this gene including splice variants and alternative transcripts. In that same previous study, we demonstrated using various outputs that *Oip5os1* is enriched in striated muscles and is upregulated in differentiating cardiac and skeletal myocytes. While *Oip5os1* is expressed in all cell types, the enrichment in cardiomyocytes and its upregulation during myocyte differentiation suggest a biological role in this cell type. We used this mouse in the current study to breed heterozygous (+/−) pairs of these mice to generate cohorts of WT (+/+) and KO (−/−) littermates that were subsequently treated with either streptozotocin (STZ) or citrate buffer (vehicle control) between 6 and 7 weeks of age, and followed for 24 weeks. The blood glucose of these animals was monitored for the following month to confirm the development of hyperglycemia (blood glucose >15 mmol/L) in STZ‐treated animals. Animals that failed to demonstrate a fasting blood glucose >15 mmol/L at 4 weeks after STZ treatment were omitted from the study. Diabetic status was further demonstrated in these mice using an oral glucose tolerance test (oGTT), which revealed elevated blood glucose of >30 mmol/L in STZ‐treated WT and KO mice across the entire 120 min testing period (Figure 1a). This is in stark contrast to citrate buffer treated mice, which demonstrated a short elevation in blood glucose (upto ~17 mmol/L) following the oral glucose bolus, which returned to baseline (~10 mmol/L) approximately 30–45 min after gavage. This elevation of blood glucose in STZ‐treated mice is consistent with fasting blood glucose (FBG) measurements performed at the end of the 24 week study, demonstrating an average FBG in STZ‐treated animals of ~28 mmol/L, while in citrate buffer treated mice we observed ~10 mmol/L (Figure 1b). There were no significant differences in any of the blood glucose parameters between WT and KO mice.

STZ‐induced diabetes leads to significant impairments in weight gain in C57BL/6J mice, with very little increase in body weight occurring in diabetic mice across the 24 week study. In comparison, non‐diabetic control mice (WT and KO) demonstrated an increase in weight from approximately 22 g up to an average of 35 g (Figure 1c). As shown in the final body weights at the end of the 24‐week period, there were no differences in body mass between WT and KO mice in either STZ or citrate buffer treated mice (Figure 1d). The major impairment in weight gain in STZ‐treated animals was observed to be related to fat mass, which was reduced from ~10 g down to ~1 g in diabetic mice (Figure 1e). There was also a significant reduction observed in lean mass of STZ animals. As above, there was no difference in the fat or lean mass between WT and KO mice in either control or diabetic setting.

### 3.2 The effects of diabetes on heart and tissue weights in *Oip5os1* WT and KO mice

**FIGURE 2:**
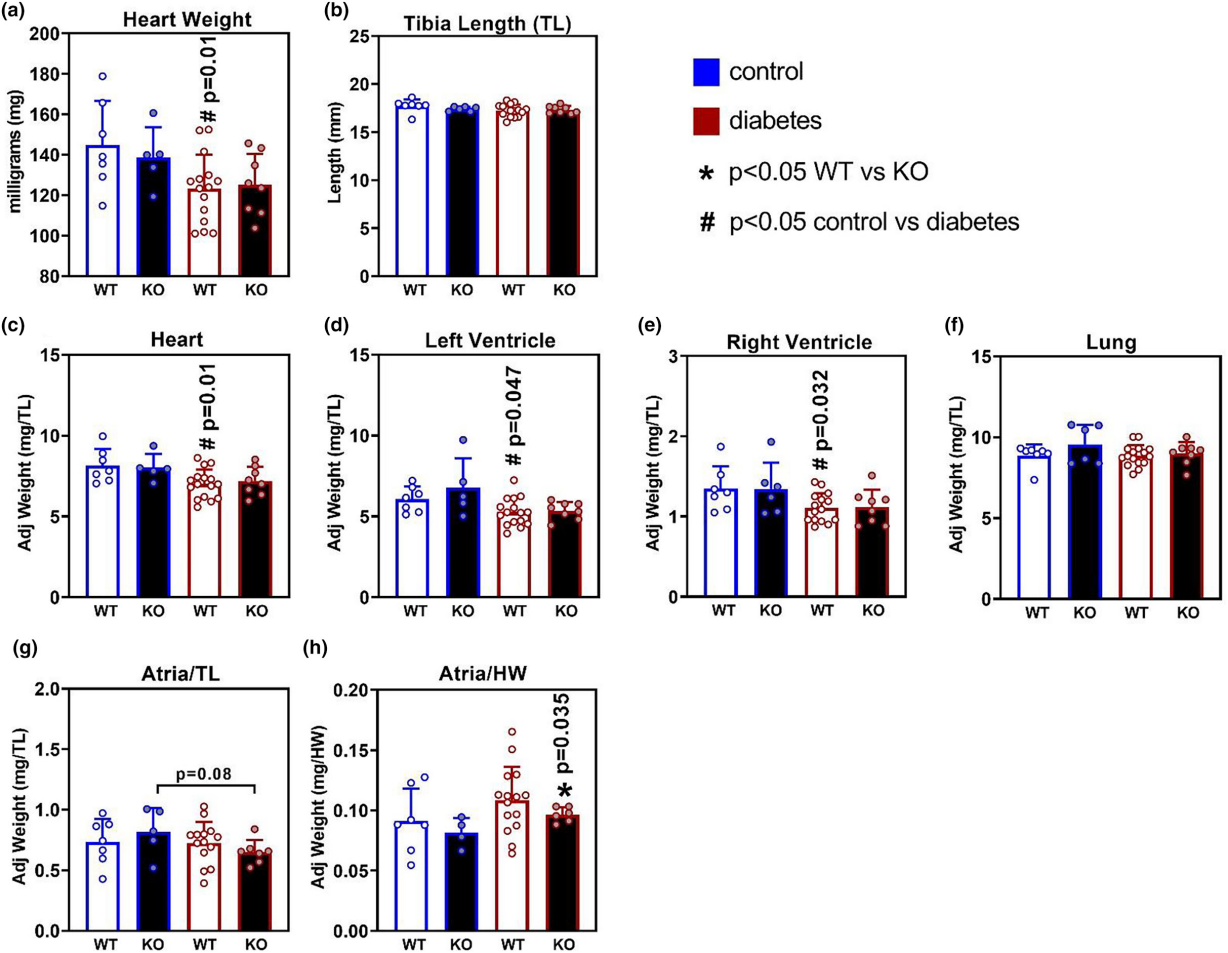
Heart and Tissue‐Specific Weights in WT and KO mice with and without diabetes. (a). Heart weights (b) and Tibia Length of each cohort at study end. Given heart weights were smaller in the setting of diabetes, region‐specific tissue weights were adjusted to tibia length. Tissue measurements as adjusted to Tibia Length (TL) as shown for (c) Heart/TL, (d) Left Ventricle/TL, (e) Right Ventricle/TL, and (f) Lung/TL. (g) Combined (left and right) atria weight adjusted for tibia length (TL) and (h) atria weight presented as adjusted (adj) for heart weight (HW) for each genotype and condition respectively. *n* = 6–15/group, data are presented as mean ± SD. BW, body weight; HW, heart weight; mg, milligram; TL, Tibia Length. * *p* < 0.05 WT versus KO, # *p* < 0.05 Control versus Diabetes as determined by unpaired *t*‐test.

To investigate the effects of *Oip5os1* deletion on heart parameters in the presence or absence of diabetes, we took several cardiac relevant measurements from animals at the end of the study. Diabetic cardiomyopathy is typically associated with diastolic dysfunction but the impact on heart weight can vary depending on the model and duration of diabetes (Bernardo et al., 2022; Chandramouli et al., 2018). Total heart weight was reduced in both WT and KO animals that had diabetes (Figure 2a). Given body weight was reduced with diabetes and tibia length was not different between WT or KO mice (Figure 2b), heart, left ventricle, right ventricle, and lung weight were normalized to tibia length. In WT diabetic mice, there was a slightly reduced normalized total heart weight (Figure 2c), left ventricle weight (Figure 2d), and right ventricle weight (Figure 2e), with no change in lung weight (a measure of pulmonary congestion secondary to heart failure) (Figure 2f). KO mice did not show any significant change in these parameters compared to diabetic mice, or to WT mice.

Further to investigating ventricle weights in these mice, we also calculated the weight of atria in each cohort. These data demonstrate that atria weight, as adjusted for tibia length, was unchanged for all groups including diabetes, although there was a trend (*p* = 0.08) for a reduction in diabetic KO atria (Figure 2g). When we adjusted atria weight to heart weight (HW) to understand atrial remodeling with diabetes or genotype independently of LV/RV parameters, we noted that this ratio was elevated in diabetic KO mice vs control KO mice, but not in diabetic WT mice (Figure 2h).

To investigate the effects of genotype and diabetes in more detail, we next analyzed functional measures of heart health and potential myopathies in these mice using echocardiography.

### 3.3 The effect of diabetes on heart function in *Oip5os1* WT and KO mice

**FIGURE 3:**
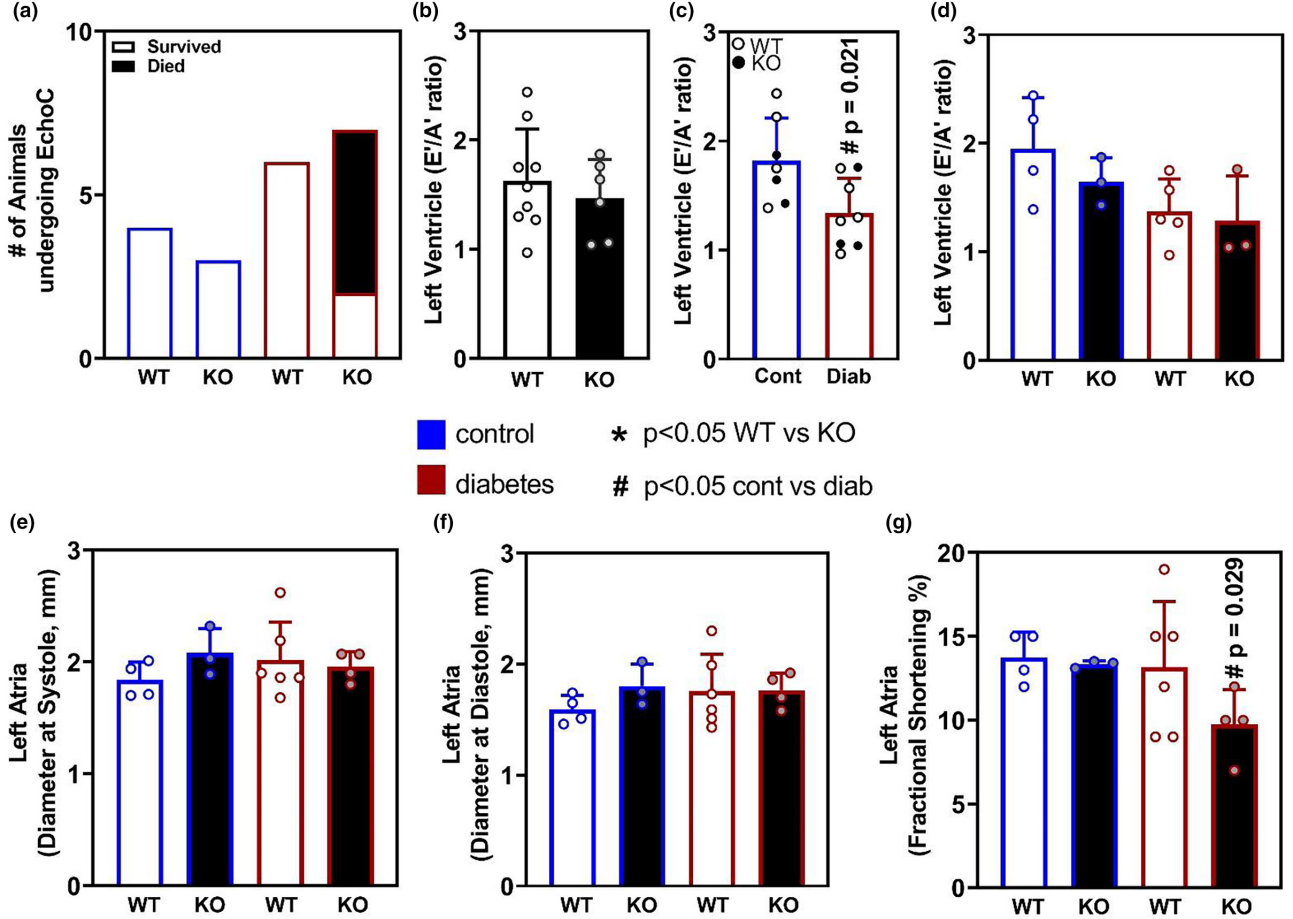
Left Ventricle and Left Atrium Function in WT and KO mice with and without diabetes. Echocardiography was performed on all four cohorts at the end of the 6‐month study. (a) Annotation of mouse groups and those that survived echocardiography or died from rapidly reducing heart rates. From mice that survived, data were analyzed and presented for Left Ventricle function including (b) E'/A' between all WT and KO mice (c) E'/A' between all Control and diabetic mice (WT = open circles, KO = closed circles) and (d) LV E'/A' between all four cohorts. Data for left atrium function were also analyzed and are presented for (e). Left Atria diameter at systole, (f) Left atria diameter at diastole, and (g) Left atria fractional shortening percentage (FS%). *n* = 3–5/group, data are presented as mean ± SD. mm = millimeters. * *p* < 0.05 WT versus KO, # *p* < 0.05 Control versus Diabetes as determined by unpaired *t*‐test.

For quantitative analysis of cardiac function in these mice, we performed echocardiography using a Vevo2100 under ketamine/xylazine anesthesia. During the echocardiography analysis, an interesting observation was revealed. Echocardiography was performed in these studies with researchers being blinded to animal genotype and treatment, and while most animals generally tolerate the anesthesia and echocardiography procedure without incident, in this study there were a subset of animals that demonstrated sudden and rapid declines in heart rate under anesthesia. Several of these animals (5/8) were unable to be recovered and succumbed to this apparent “intolerance” to anesthesia. Upon unblinding of the data, it was found that all of the mice that demonstrated anesthesia intolerance were diabetic *Oip5os1* KO mice (Figure 3a). No animals from any of the other genotype groups demonstrated a similar reduction in heart rate, or succumbed to these effects, indicating a specific effect in diabetic KO animals. Because functional data could not be collected from some mice due to this intolerance, mouse numbers were lower than planned in KO animals with diabetes, reducing statistical power.

Nevertheless, to understand if genotype and diabetes impacted cardiac dysfunction and contributed to the intolerance effects above, we analyzed echocardiography data for left ventricle (LV) and left atrium (LA) function. This included diastolic outputs and fractional shortening in LA, where all parameters analyzed are presented in Table 2. Because diabetes is often associated with diastolic dysfunction in the LV, we first sought to investigate whether genotype, diabetes or a combination of these interventions led to changes in cardiac function. We demonstrated that genotype alone across both treatment groups did not have any significant impact on LV E'/A' ratio under control conditions (Figure 3b). However, when we analyzed all groups together for the effects of diabetes, we demonstrate that mice with diabetes had a significantly (*p* = 0.041) reduced LV E'/A' ratio compared to control animals (Figure 3c); this was independent of genotype with clear overlap between WT and KO mice under control or diabetic conditions. When we analyzed the four groups independently, WT and KO mice with and without diabetes, it was shown that no group was statistically different from the others, although diabetes did demonstrate a trend (*p* = 0.059) to reduce E'/A' in WT mice (Figure 3d). There were also significant changes in other parameters that are used to calculate E'/A', mostly relating to the A‐wave parameters (Table 2). With regard to left atrium function, we demonstrate that there were no significant differences between LA dimension at systole (Figure 3e) or diastole (Figure 3f); however, when fractional shortening (FS) was calculated in the LA, there was a significant reduction (~30%) in FS% in *Oip5os1* KO diabetic mice (Figure 3g). With regard to understanding this finding specifically, it is important to consider that FS% in the LA of a healthy mouse is generally ~10%–15% (i.e., the diameter only shortens by ~15% during contraction, compared to up to 40% in LV). As shown in Table 2 in the manuscript, the FS% changed from 13.3% in KO Cont animals to 9.9% in KO Diab mice. The KO Diab mice therefore have ~70% of the function of a KO Cont animal, equating to a ~ 30% reduction. This result suggests a reduced contractile capacity in the LA of these mice compared to non‐diabetic KO mice, and a similarly reduced trend compared to WT diabetic mice. This reduced contractile function is consistent with the significantly increased combined atrial weight observed in KO mice, suggestive of a myopathy‐like phenotype.

**TABLE 2:** Echocardiography data.

	Left ventricle function							
	Heart rate	A'	E'	MV A	MV E	E'/A'	MV E/A	MV E/E'
	BPM	mm/s	mm/s	mm/s	mm/s	–	–	–
WT Cont, *n* = 4	376 ± 8	−13.4 ± 0.9	−25.8 ± 2.7	392 ± 45.3	727 ± 36.7	1.95 ± 0.24	1.91 ± 0.18	−28.9 ± 2.5
WT Diab, *n* = 6	386 ± 8	−17.3 ± 1.9	−23.5 ± 3.1	468 ± 28.1	753 ± 33.6	1.37 ± 0.14	1.62 ± 0.07	−33.8 ± 4.2
KO Cont, *n* = 3	396 ± 13	−15.1 ± 1.5	−25.3 ± 4.3	382 ± 30.8	717 ± 28.3	1.64 ± 0.13	1.89 ± 0.16	−29.8 ± 4.3
KO Diab, *n* = 3	374 ± 12	−19.0 ± 0.9	−24.4 ± 4.4	435 ± 14.6	769 ± 42.2	1.28 ± 0.24	1.76 ± 0.05	−32.9 ± 3.8
*Group Stats*								
*WT versus KO*								
*Cont versus Diab*		*		*		*		

	Left Atria Function			
	Heart rate	LA_s	LA_d	LA FS
	BPM	mm	mm	%
WT Cont, *n* = 4	404 ± 15	1.84 ± 0.08	1.59 ± 0.06	13.6 ± 0.7
WT Diab, *n* = 6	397 ± 7	2.02 ± 0.14	1.76 ± 0.13	13.2 ± 1.5
KO Cont, *n* = 3	433 ± 14	2.08 ± 0.13	1.80 ± 0.11	13.3 ± 0.1
KO Diab, *n* = 3	373 ± 22	1.96 ± 0.07	1.77 ± 0.08	**9.9 ± 1.1#**
*Group Stats*				
*WT versus KO*				
*Cont versus Diab*				

Given the above‐observed changes in functional parameters across different chambers in the heart with genotype and diabetes, we sought to determine whether there were changes in gene expression in these tissues that might support and explain these findings.

### 3.4 The effect of diabetes and genotype on left ventricle (LV) gene expression

**FIGURE 4:**
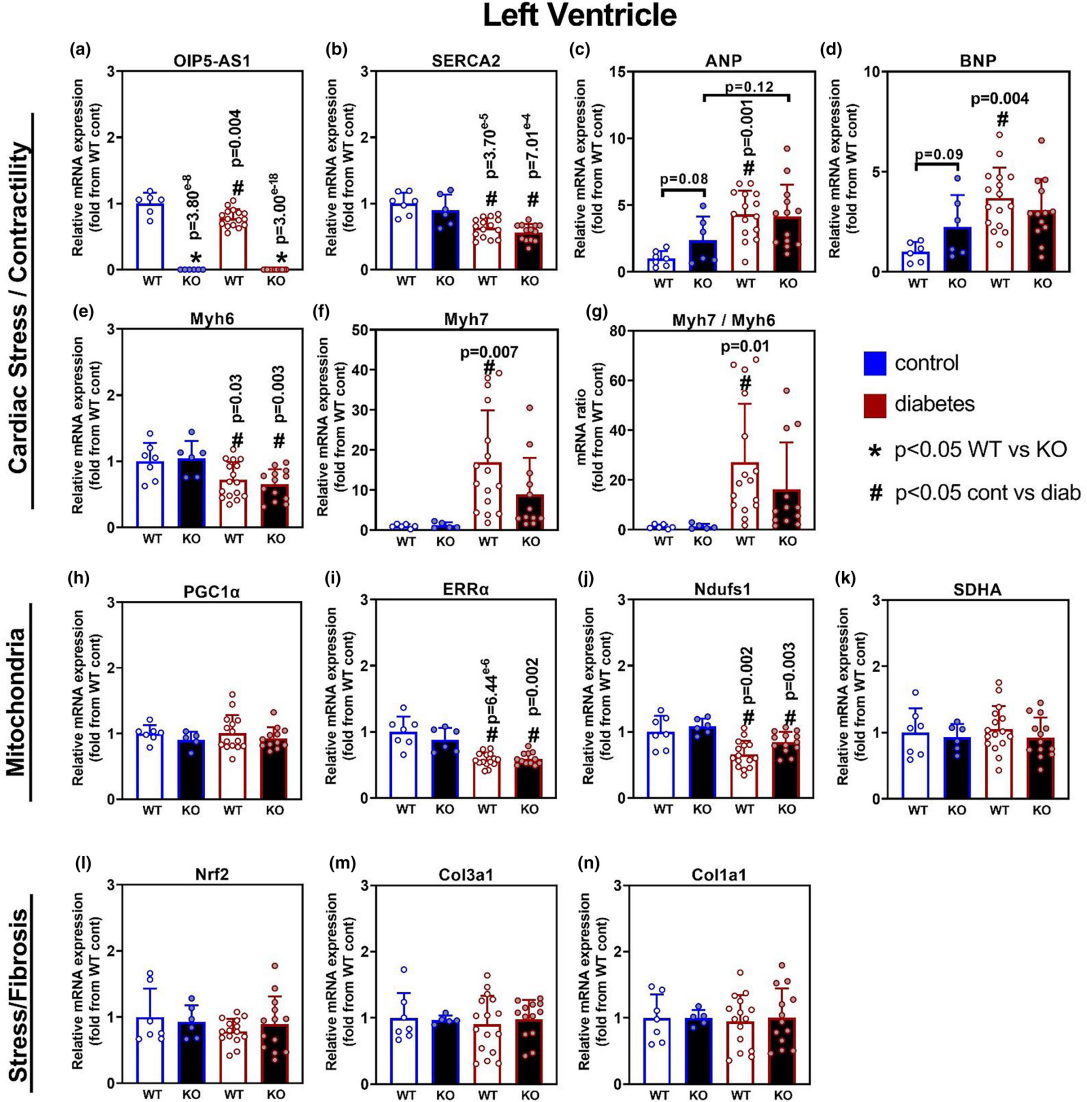
Gene expression Analysis in Left Ventricle in WT and KO mice with and without diabetes. Gene expression was determined by qPCR for various molecular pathways including cardiomyopathy, mitochondrial function, and stress/fibrosis across the four cohorts. Genes for cardiac stress and contractility included (a) *Oip5os1*, (b) SERCA (*Atp2a2*) (c) ANP (*Nppa*), (d) BNP (*Nppb*), (e) *Myh6*, and (f) *Myh7*. (g) *Myh7/Myh6* mRNA ratio. Genes for mitochondrial function included (h) PGC1α (*Ppargc1a*), (i) ERRα (*Esrra*) (j) *Ndufs1*, and (k) *Sdha*. Genes for Stress/Fibrosis included (l) *Nrf1*, (m) *Col3a1* (procollagen) and (n) *Col1a1*. *n* = 6–15/group, data presented as mean ± SD. *p < 0.05 WT versus KO, # *p* < 0.05 Control versus Diabetes as determined by unpaired *t*‐test.

Diabetic cardiomyopathy is typically associated with changes in cardiac stress and contractile related genes. Here, we assessed the impact of genotype and diabetes on gene expression in the LV by qPCR. Data demonstrated that as expected, there was no detectable expression of *Oip5os1* in both KO cohorts (Figure 4a). Of note, in previous work our group described a reduction in the expression of *Oip5os1* in WT mice in the setting of pressure overload‐induced heart failure (Zhuang, Calkin, et al., 2021). Here, we observed a significant ~40% reduction in expression of *Oip5os1* in WT mice in the setting of diabetes, highlighting a conserved effect of a cardiac stress on *Oip5os1* expression in mice. Consistent with a cardiomyopathy phenotype being present in these mice with diabetes, we observed significant reductions in the expression of *Atp2a2 (SERCA2)* (Figure 4b), and significant or strong trends for changes in other classic cardiac stress and contractile genes including *Nppa* (ANP) (WT: *p* < 0.05, KO: *p* = 0.12) (Figure 4c), *Nppb* (BNP) (WT: *p* < 0.05) (Figure 4d), *Myh6* (WT and KO: *p* < 0.05) (Figure 4e), and *Myh7* (WT: *p* < 0.05, KO: *p* = 0.08) (Figure 4f). Moreover, when we analyzed the ratio of *Myh7* / *Myh6* expression, a common ratio investigated in cardiomyopathy readouts, we observed between 15 and 20 fold differences in diabetes, which was significant in WT mice and trending in KO animals (WT: *p* < 0.05, KO: *p* = 0.1) (Figure 4g). With regard to genotype, we did not observe any remarkable changes in expression of these HF markers in KO mice compared to WT, either with or without diabetes, consistent with there being no significant change in LV functional readouts as determined by echocardiography.

In our previous work investigating the role of *Oip5os1* in pressure overload‐induced cardiac pathology, we observed changes in several genes linked to mitochondrial function, which might explain the worsened phenotype in KO mice. Here, we investigated genes in the setting of diabetic cardiomyopathy and demonstrate that there was a consistent effect of diabetes on mitochondrial gene expression signatures in the LV. Specifically, while we did not observe any differences in the mitochondrial transcription factor *Ppargc1a* (PGC1α) (Figure 4h), there were reductions in the nuclear receptor *Esrra* (ERRα) (Figure 4i) and a core protein of Complex I from the mitochondrial electron transport chain (ETC), *Ndufs1* (Figure 4j). There were no changes observed in a core protein from Complex II of the ETC (Figure 4k). Genotype had no effect on any of these genes. With regard to stress and fibrosis pathways, analysis of key genes related to these pathways was unchanged with either diabetes or genotype, including *Nrf2* (Figure 4l), procollagen (Figure 4m), and *Col1a1* (Figure 4n), suggesting that LV fibrosis is not a key feature contributing to diastolic dysfunction in this model. Overall, diabetes had a detectable effect on cardiac and metabolic genes in the LV that were consistent with cardiomyopathy; however, *Oip5os1* KO did not influence these outcomes.

### 3.5 The effect of diabetes and genotype on right ventricle (RV) gene expression

**FIGURE 5:**
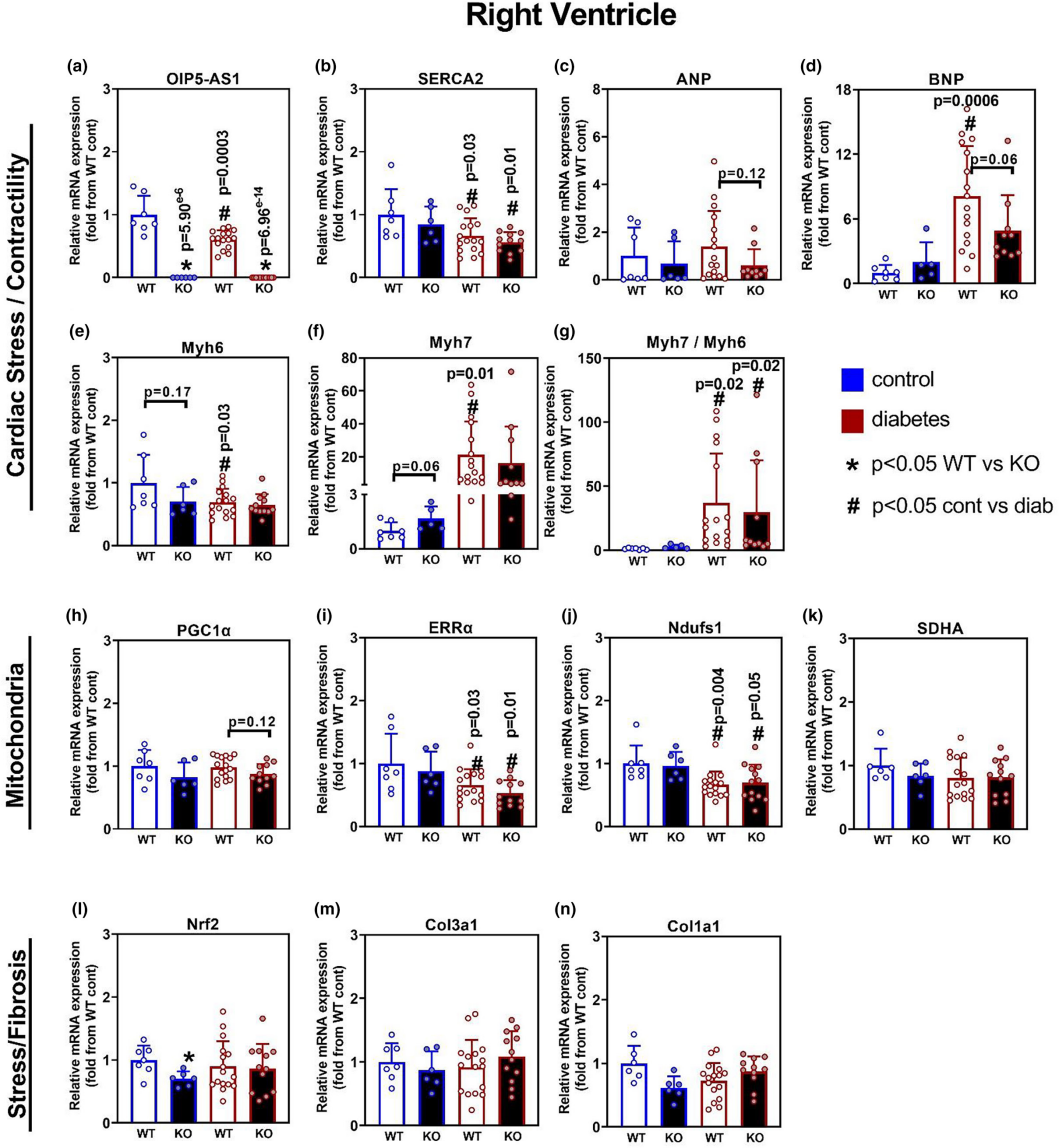
Gene expression Analysis in Right Ventricle in WT and KO mice with and without diabetes. Gene expression was determined by qPCR for various molecular pathways including cardiomyopathy, mitochondrial function, and stress/fibrosis across the four cohorts. Genes for cardiac stress and contractility included (a) *Oip5os1*, (b) SERCA (*Atp2a2*) (c) ANP (*Nppa*), (d) BNP (*Nppb*), (e) *Myh6*, and (f) *Myh7*. (g) *Myh7/Myh6* mRNA ratio. Genes for mitochondrial function included H. PGC1α (*Ppargc1a*), (i) ERRα (*Esrra*) (j) *Ndufs1*, and (k) *Sdha*. Genes for stress/fibrosis included (l) *Nrf1*, (m) *Col3a1* (procollagen), and (n) *Col1a1*. *n* = 6–15/group, data presented as mean ± SD. *p < 0.05 WT versus KO, # *p* < 0.05 Control versus Diabetes as determined by unpaired *t*‐test.

With the observation that LV of mice with diabetes demonstrated hallmarks of diabetic cardiomyopathy at both the functional and molecular level, we investigated further if the right ventricle (RV) also displayed similar traits, or indeed was impacted differentially with regard to genotype. Consistent with LV data, *Oip5os1* was not detectable in KO animals, and diabetes similarly reduced the expression of *Oip5os1* significantly by ~40% in WT mice (Figure 5a). Also consistent with LV data was the reduction in SERCA2a expression with diabetes (Figure 5b), but no changes were observed with genotype. Interestingly, when analyzing genes related to cardiac stress and contractility in RV, the pattern of expression was slightly different to that observed in the LV. Of particular note was the trend for reduced activation of *Nppa* (ANP) and *Nppb* (BNP) expression to diabetes in KO animals (*p* = 0.12 and *p* = 0.06, respectively) (Figure 5c,d). However, the *Myh6* and *Myh7* genes were mostly comparable between WT and KO animals with diabetes (Figure 5e,f), with the *Myh7/Myh6* ratio demonstrating a significant increase by ~20–30 fold in both WT and KO mice with diabetes (Figure 5g). These genotype effects were mostly not observed in the LV of the same mice, indicating chamber‐specific effects of diabetes on these parameters.

With regard to mitochondrial and stress/fibrosis gene signatures in the RV, we observed similar patterns of expression to that shown above in the LV (Figure 5h–m). In particular, ERRα (Figure 5i) and Ndufs1 (Figure 5j) were significantly reduced in diabetes compared to control animals, as observed in the LV. Fibrosis readouts were also not changed in the setting of diabetes in the RV (Figure 5m,n) similarly to LV, again supporting the notion that fibrosis does not overtly contribute to dysfunction driven by diabetes in our STZ model (at least within 6 months).

### 3.6 The effect of diabetes and genotype on atrial gene expression

Echocardiography data demonstrated a significantly reduced fractional shortening (%FS) in the left atrium (LA), indicating a potential genotype by intervention (diabetes) effect on heart function in this chamber. To investigate this further, we performed the same molecular analysis using qPCR as described above for the LV and RV.

**FIGURE 6:**
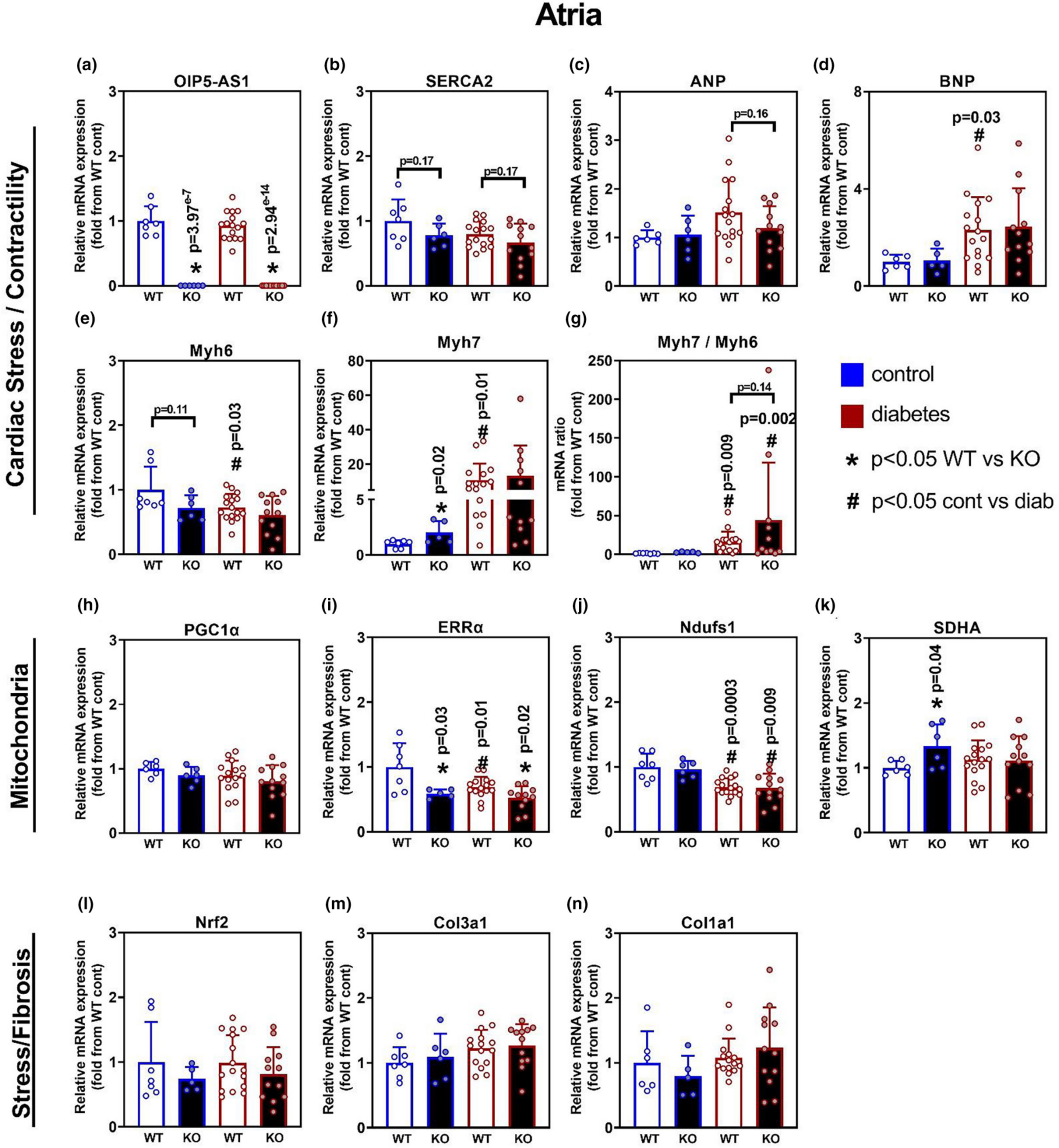
Gene expression Analysis in Atria (combined) in WT and KO mice with and without diabetes. Gene expression was determined by qPCR for various molecular pathways including cardiomyopathy, mitochondrial function, and stress/fibrosis across the four cohorts. Genes for cardiac stress and contractility included (a) *Oip5os1*, (b) SERCA (*Atp2a2*) (c) ANP (*Nppa*), (d) BNP (*Nppb*), (e) *Myh6*, and (f) *Myh7*. (g) *Myh7/Myh6* mRNA ratio. Genes for mitochondrial function included H. PGC1α (*Ppargc1a*), (i) ERRα (*Esrra*), (j) *Ndufs1*, and (k) *Sdha*. Genes for stress/fibrosis included (l) *Nrf1*, (m) *Col3a1* (procollagen), and (n) *Col1a1*. *n* = 6–15/group, data presented as mean ± SD. * *p* < 0.05 WT versus KO, # *p* < 0.05 Control versus Diabetes as determined by unpaired *t*‐test. Of note, once it was realized that KO diabetic mice were not surviving the echocardiography procedure it was not ethical to continue with cardiac function assessment, but atria were collected from these mice to increase the power of identifying any molecular differences.

qPCR data acquired from the atria tissue presented in a relatively different pattern of expression compared to the same analysis performed in ventricle tissue. Firstly, while *Oip5os1* was undetectable in KO tissues, its expression was not reduced with diabetes in WT mice as was observed in both RV and LV (Figure 6a). Moreover, the contractile responsive gene SERCA2a, which was consistently reduced with diabetes in both LV and RV tissues, was also unaffected by diabetes in the atria (Figure 6b). Interestingly however, SERCA2a expression demonstrated a strong trend (both *p* = 0.17) toward being reduced in KO atria tissue, providing evidence for a dysfunction like phenotype in the atria driven by genotype, consistent with echocardiography derived %FS data (Figure 3f). There were no significant changes in the expression of *Nppa* (ANP) in diabetes (Figure 6c), but a slight increase in *Nppb* (BNP) (Figure 6d), which was significant in WT mice. Significant differences or trends in *Myh6* and *Mhy7* with diabetes and genotype (Figure 6e,f) translated into a significant increase in the *Myh7 / Myh6* ratio with diabetes in both genotypes, with a trend for a greater increase in KO mice (*p* = 0.14) (Figure 6g).

With regard to mitochondrial gene expression patterns in atria, consistent with LV/RV data there was again no change in PGC1α (Figure 6h). ERRα expression was comparably reduced in atria of WT diabetic mice and both control and diabetic KO mice (Figure 6i). *Ndufs1* expression was reduced with diabetes as seen in the LV and RV (Figure 6j), and *Sdha* appeared to be elevated mildly but significantly in KO non‐diabetic atria (Figure 6k). There were no effects on the expression of stress/fibrosis genes with either genotype or diabetic status (Figure 6l–n).

## 4 DISCUSSION

In the current study, we investigated the role of the lncRNA *Oip5os1* in STZ‐induced diabetic cardiomyopathy and heart dysfunction. We have previously demonstrated that a KO mouse model of this lncRNA demonstrated worsened heart failure in a pressure overload model (Zhuang, Calkin, et al., 2021). Thus, further characterization of *Oip5os1* in various pathological scenarios provides a deeper understanding of the unique functions of this lncRNA in striated muscles including the heart. The most striking observation in the current study was atria dysfunction of diabetic KO mice. Understanding and defining molecular mechanisms underlying different forms of atrial myopathy and atrial dysfunction is considered critical for preventing and treating atrial pathology including atrial fibrillation (Chen et al., 2021). Here, we demonstrate a role for *Oip5os1* protecting against atrial dysfunction in a setting of type 1 diabetes‐induced cardiomyopathy. Left atrial (LA) dysfunction has been identified in children and young children with type 1 diabetes (Ifuku et al., 2021). Further, diabetes is a known risk factor for atrial fibrillation, and patients with diabetes have been shown to have an increased risk of atrial fibrillation (~15% vs. 1%–4% in the general population) (De Sensi et al., 2015; Dublin et al., 2010; Ugowe et al., 2019).

Our functional and molecular analysis of this model was consistent with diabetes leading to cardiomyopathy associated with diastolic dysfunction, as described previously in other studies (Prakoso et al., 2017, 2020; Ritchie et al., 2012). Specifically, we observed differences in left ventricle diastolic function via reductions in E'/A' ratio, but did not observe genotype by diabetes impacts (i.e., KO diabetic animals were not different to WT diabetic animals). We also observed that diabetes led to a reduction in the expression of *Oip5os1* in ventricle tissue, associating the expression of this lncRNA to diabetic cardiomyopathy. Changes in *Oip5os1* expression in ventricle tissue from diabetic animals was accompanied by changes in classic cardiac stress “neonatal” gene expression signatures including increases in *Nppa* (ANP) and *Nppb* (BNP) expression, as well as reductions in *Myh6* and increases in *Myh7*; highlighted by an increase in the *Myh7/Myh6* ratio. Moreover, we observed consistent reductions in SERCA2a, another well‐described molecular consequence and potential driver of contractile dysfunction and heart failure. Nevertheless, while we confirm that diabetes has an impact on LV function and molecular markers, we did not observe any impact of genotype on this response indicating that *Oip5os1* was likely not worsening heart function outcomes in LV tissue in the setting of diabetes.

With the lack of genotype effect in LV of these mice, we revisited our prior *Oip5os1* KO data from the setting of pressure overload, to investigate if other more subtle phenotypes might provide insight for the current study. In our previous work, while pressure overload is a more severe model and led to changes in LV function, we did see evidence for the KO of *Oip5os1* having impacts in other chambers of the heart including RV and atria (Zhuang, Calkin, et al., 2021). Accordingly, we investigated whether KO and diabetes might also impact these tissues in the current model. While there were similar molecular changes in the RV of diabetic mice, there were no differences between WT and KO in the RV. However, when we investigated left atrial (LA) function by echocardiography, we noted a significant reduction in fractional shortening in the LA of diabetic KO mice, which was accompanied by a significant increase in atrial weight relative to total heart weight—perhaps indicating myopathy and dysfunction. Unfortunately, the number of mice analyzed in these experiments was low due to the increased mortality rate in KO diabetic animals (Figure 3a), which was made more difficult due to Echo data in this study being focused on LV function, and not LA function—hence some parameters in various mice were unable to be analyzed. Nevertheless, despite these technical challenges, the data still demonstrated a significant effect in KO diabetic animals on LA function.

When we analyzed the qPCR data from atria tissue, we observed trends for a reduction in SERCA2a in KO animals compared to WT animals regardless of diabetes status and also observed a more robust shift in the Myh7/6 ratio—both consistent of a more severe atrial dysfunction in KO diabetic mice. These changes were interesting in the context of our prior pressure overload studies, because one of the most striking clinical features on those mice was the substantial increase in atrial size, and overt clotting in the atrial appendage. Collectively, our data across the two different heart intervention models suggest that *Oip5os1* may have a specific impact on atria functionality.

With regard to the underlying mechanisms that may have precipitated such a phenotype, we observed changes in gene expression in both ventricles and atria tissue that suggest an alteration in the molecular regulation of mitochondrial function. These included significant changes in the expression of ERRα in KO atria, which has been shown previously to be important for heart development via its effects on mitochondrial function (Wang et al., 2015). In our previous work, we demonstrated that loss of *Oip5os1* led to reductions in ERRgamma, and other genes involved in mitochondrial function including genes relating to Complex I (*Ndufs1*) and PGC1α. ERRgamma has been shown to impact on heart development and metabolism via its effect on mitochondrial function (Alaynick et al., 2007; Wang et al., 2015). The reduction in ERRα in the atria in our current study may indicate a consistent impact of *Oip5os1* on the Estrogen Receptor Related protein family that ultimately leads to dysfunction in mitochondrial molecular pathways and worsened heart function. This worsened “function” was supported by the change in expression of cardiomyopathy related transcripts as shown above, and is consistent with read outs of heart failure in humans (Sihag et al., 2009).

In general, the findings from our current work are in agreement with our prior studies demonstrating a worsened cardiac function with KO of *Oip5os1*, and of those by others that have demonstrated that overexpression of *Oip5os1* was protective against ischemia reperfusion injury in rodent models (Niu et al., 2020). Interestingly, this group also proposed that this protection was mediated in part by improvements in mitochondrial function, linking *Oip5os1* activity with mitochondria and heart function in three separate studies. The specific mechanistic underpinnings of such an effect may be related to the previously described mechanism of action of *Oip5os1*, which has mostly been related to its ability to act as a molecular sponge against miRNA7, which impacted the abundance of several other RNAs (Arunkumar et al., 2018; Jones et al., 2020; Yang et al., 2022). Given that *Oip5os1* is not particularly enriched differentially between the different chambers of the heart, it is possible that its molecular targets including miRNA7 may well be chamber specific and therefore explain the differential responses.

Nevertheless, while our data are suggestive of a role for *Oip5os1* in left atrial function, there are some limitations to the interpretation in this study. An important consideration not able to be studied in this current work is the impact of biological sex. We demonstrated in our prior work that female KO mice were more prone to LV heart failure following pressure overload. In our current study using STZ, we were limited to using males because of the previously well‐described protection against STZ‐induced diabetes (at 55 mg/kg) that females are afforded (Le May et al., 2006). While diabetes induction may be successful in females when using higher doses of STZ, it would introduce other complex variables in terms of off‐target effects of STZ that may also impact heart function, that therefore preclude the direct comparison with outcomes in males. Thus, in future studies it would be interesting to investigate the impact of diabetes, perhaps induced by other means such as genetics, on LA function in female mice. Another limitation is the reduced numbers, and consequently statistical power in readouts of heart function, mostly related to the loss of animals under anesthesia. While this was a limitation (albeit still demonstrating significant changes in some parameters), it also raised interesting questions as to what was driving this major intolerance to anesthesia in the diabetic KO animals. These animals were not smaller compared with control diabetic mice, not determined to be unwell, and also had no indication of an elevated inflammatory or immune response. Therefore, we can only conclude from these data that these mice likely succumbed to dysfunction arising from poor LA function.

In summary, our current work sheds further light on our understanding of *Oip5os1* in the setting of heart disease, in this instance precipitated by STZ‐induced diabetes. Our findings suggest a specific effect of this lncRNA in the atria of mice, which are consistent with other studies that have demonstrated an effect of *Oip5os1* deletion in striated muscles. The association of this lncRNA with mitochondrial function provides further evidence for these pathways to be important in the development of cardiac pathology, and provide additional evidence for a functional role of lncRNAs as being important mediators of cardiac outcomes.

## AUTHOR CONTRIBUTIONS

BGD and AZ conceived and designed the study. BGD wrote the manuscript. AZ, YT, YL, CY, HK, KG, and SW generated datasets, analyzed data, and interpreted research findings. AZ, YT, YL, SW, and STB performed animal experiments and analyzed data. KG and HK performed, analyzed or interpreted echocardiography data. BGD, ACC, and JRM provided resources, data analysis, experimental guidance, and reagents. All authors had the opportunity to agree to the content and read, edit and provide feedback on the submitted manuscript.

## CONFLICT OF INTEREST STATEMENT

The authors declare they have no conflict of interest.

## ETHICS STATEMENT

All experiments were approved by the Alfred Research Alliance (ARA) Animal Ethics Committee and performed in accordance with the NHMRC of Australia guidelines for the care and use of laboratory animals.
